# Safety of insecticide-treated mosquito nets for infants and their mothers: randomized controlled community trial in Burkina Faso

**DOI:** 10.1186/s12936-015-1068-6

**Published:** 2015-12-30

**Authors:** Guangyu Lu, Corneille Traoré, Peter Meissner, Bocar Kouyaté, Gisela Kynast-Wolf, Claudia Beiersmann, Boubacar Coulibaly, Heiko Becher, Olaf Müller

**Affiliations:** Institute of Public Health, Medical School, Ruprecht-Karls-University, INF 324, 69120 Heidelberg, Germany; Commission de l’UEMOA, Direction de la Santé, de la Protection Sociale et de la Mutualité, Ouagadougou, Burkina Faso; Department of Pediatric and Adolescent Medicine, Ulm University, Ulm, Germany; Centre de Recherche en Santé de Nouna (CRSN), Nouna, Burkina Faso; Institute of Medical Biometry and Epidemiology, University Hospital Hamburg-Eppendorf, Hamburg, Germany

**Keywords:** Malaria, Insecticide treated bed net (ITN), Synthetic pyrethroids, Safety, Side effects, Burkina Faso

## Abstract

**Background:**

Insecticide-treated bed nets (ITNs) are now the main tool for malaria prevention in endemic areas. Synthetic pyrethroids are the only group of insecticides recommended by the World Health Organization for the use on ITNs. There are only few studies which have specifically investigated potential adverse effects of frequent exposure to ITNs in the vulnerable group of young infants and their mothers.

**Methods:**

This study was nested into a large randomized controlled ITN effectiveness trial. Ninety newborns and their mothers were selected from the study population for participation. Together with their mothers they were protected with ITNs from birth (group A, n = 45) or from age 6 months (group B, n = 45) and followed up for 18 weeks (daily visits in the first 4 weeks, weekly visits thereafter). Potential side effects related to synthetic pyrethroids (deltamethrin) exposure were systematically investigated by trained field staff. The frequency and duration of respective symptoms was compared between the two study groups.

**Results:**

A total of 180 participants (90 mothers and 90 infants) were followed up over the study period without any loss to follow up. There were no significant differences in the frequency and duration of side effects between the two study groups, except that the frequency of headache was significantly higher in group A compared to group B mothers (p = 0.01).

**Conclusions:**

The study provides further evidence for ITNs being sufficiently safe in children and even in newborns. The association with headache in mothers could be explained by them handling the ITNs more intensely or it could be a chance finding.

## Background

Although malaria control interventions have been substantially scaled up during the past decade malaria still accounts for an estimated 190 million clinical cases and 584,000 deaths every year [1]. The majority of severe cases and deaths occur in sub-Saharan Africa (SSA), mainly in pregnant women and young children of remote rural areas [2]. Insecticide-treated bed nets (ITNs) are considered the most important tool for malaria prevention in endemic areas and were found to be highly effective in reducing malaria morbidity and mortality in many different epidemiological settings [2, 3]. Therefore, scaling up ITN coverage and usage by young children and pregnant women is among the major targets of the international efforts to roll back malaria [4–6].

Synthetic pyrethroid insecticides were mainly developed by the team of Michael Elliott at Rothamsted Research in the 1960s and 1970s [7]. Compared to natural pyrethrin, synthetic pyrethroids are much more stable and have, therefore, been widely used in agriculture to control a variety of pests [8]. Investigations on the potential of ITNs to control malaria started during the 1980s [2, 9]. However, decades later, synthetic pyrethroids remain the only group of insecticides approved from the World Health Organization Pesticide Evaluation Scheme (WHOPES) to be used for treating mosquito nets [10]. This is because of the rapid knock-down effects and high insecticidal potency of pyrethroids, combined with their relative safety for human contact and domestic handling [2, 10, 11]. Pyrethroids are evoking no major symptoms of poisoning if used with the recommended precautions; only mild and reversible paraesthesia has regularly been reported from persons having been in unprotected contact with the insecticide or with ITNs [12–14]. However, some debate on the safety of frequent exposure to low concentrations of pyrethroids continued, especially after evidence for an irreversible and cumulative effect of pyrethroid on nerve tissue in animal models was published in 1984 [15, 16]. As a consequence, market withdrawal has even been considered in some developed countries until all adverse effects could be ruled out [17, 18]. Moreover, occupational poisoning from pyrethroids among the people doing the spraying was reported from some countries like China [19–22]. However, only very little data are available on potential toxicity of frequent or long-term exposure to low-level pyrethroids among ITNs users in malaria endemic countries [14, 15, 23].

Among the ITN users, infants are considered biologically more vulnerable and thus likely more susceptible for insecticide side effects, which may be aggravated by oral exposure [24–26]. It has been observed that infants frequently suck and chew ITNs which may accumulate insecticide in their body. Moreover, young infants are likely more susceptible to the neurotoxic effects of synthetic pyrethroids compared to older children [15, 16, 26].

This study presents data which were prospectively collected from a large randomized controlled trial (RCT) on the effects of deltamethrin among infant-mother-pairs in a malaria endemic area of rural Burkina Faso.

## Methods

### Study area

The trial took place in the rural research zone of the Centre de Recherche en Santé de Nouna (CRSN) in Nouna Health District (NHD), north-western Burkina Faso [27]. The Nouna area is a dry orchard savannah, populated mainly by subsistence farmers of various ethnic groups [28].

Malaria was holoendemic but highly seasonal in the study area; the rainy season lasts from July until October and the main transmission period is between July and December [29]. The number of infective bites per person per year-i.e. the Entomological Inoculation Rate (EIR)—varied between 100 and 1000 in the different study villages [30]. ITNs were not available in the study area until the start of this trial in the year 2000, but about one quarter of young children were usually protected with untreated bed nets in the study villages during rainy seasons [27]. Mothers regularly sleep with infants under the same bed net.

### Study design and participants

This study was nested into a large randomized controlled effectiveness trial [27]. In brief, a total of 3387 neonates from all 41 villages of the rural CRSN study area were individually randomized to receive either ITN protection from birth (group A) or from 6 months of age (group B) until their fifths birthday. Malaria incidence and all-cause mortality were the primary endpoints.

A convenience sample of 90 neonates (45 group A, 45 group B) and their mothers from five representative villages of the CRSN study area were selected for this survey. In these five villages, the first children recruited for the trial were subsequently included into this sub-study until the overall sample size was reached. Inclusion criteria were recruitment within 2 weeks of birth, and being a permanent resident in the study villages. Recruitment was done by fieldworkers who were regularly informed about births by village informants during their twice-weekly visits to the study villages [27].

First generation PermaNet™, a product of Vestergaard Frandsen Group in Denmark, was used during this trial [27]. These ITNs were family size rectangular green nets of 100 denier strength [31]. Study children and mothers were visited by village-based trained field staff daily in the first 4 weeks and then followed up weekly for another 14 weeks, so the total follow-up period was 18 weeks. A questionnaire administered to the mothers during visits inquired about the presence and duration of side effects potentially caused by pyrethroids in both the mothers and the infants (e.g. pruritus,conjunctivitis,skin eruption, headache/dizziness, paresthesia). Field workers were trained to verify the reported symptoms through direct observation/examination if possible.

### Data management and analysis

Data were cleaned and entered by the field staff of the trial in collaboration with the data management center of CRSN [27]. Data analysis based on the original database of the trial took place at the University of Heidelberg. The frequency of infants or mothers with specific symptoms (defined as at least one episode of respective side effects) and the duration of symptoms in days of potential side effects was compared for different follow up periods (the first week, the 2nd to the 4th weeks and the total 18 weeks).

Statistical analysis was performed with SAS software (version 9.2, 2015). Fisher’s Test was used to compare differences in frequency of symptoms between group A and B according to the different follow up periods. Risk ratios with 95 % exact confidence intervals (CI) were calculated on the frequency of each side effect. The Wilcoxon Rank Sum Test was used to compare differences in the duration of the symptoms between group A and group B according to the different follow up periods. Significance was accepted at p ≤ 0.05.

### Ethical aspects

The study protocol was approved by the Ethical Committee of the Heidelberg University Medical School and the local Ethical Committee in Nouna, Burkina Faso. Informed community consent was sought through explanations and discussions during village meetings before the study was implemented. Oral informed consent from interviewees was a prerequisite for the interviews and the observations/examinations.

## Results

All trial children of this sub-study were followed up from January, 2001 until April, 2002, without any loss to follow-up. The percentage of follow-up visits without records is around 10 % for the total follow-up period, with no significant differences between the two study groups.

### Infants

The associations between pyrethroid exposure and the occurrence of the reported side effects are shown in Table 1. The most frequently mentioned symptoms of infants were rhinitis and cough. In addition, side effects, such as skin eruption diarrhoea, conjunctivitis, vomiting, anal fissure and others were reported; other (rare) symptoms include mycoses, ear pain, convulsion, open wound, inflammation, stomach ache and constipation. There were no significant differences in the frequency and duration of these symptoms between the two study groups during the overall follow up.

**Table 1:** Frequency and duration of reported side effects among infants of group A (N = 45) and group B (N = 45) of an ITN trial conducted in Burkina Faso

Reported side effects	Week 1							Week 2–4							Week 1–18						
	Frequency^a^		RR (95 % CI)^a^	P-value^a^	Duration of symptom (Days)		P-value^c^	Frequency		RR (95 % CI)	P- value	Duration of symptom (Days)		P-value	Frequency		RR (95 % CI)	P-value	Duration of symptom (Days)		P-value
	A	B			A	B		A	B			A	B		A	B			A	B	
Rhinitis	10	9	1.14 (0.37–3.60)	1.00	41	40	0.87	16	13	1.36 (0.51–3.64)	0.65	151	98	0.53	20	19	1.09 (0.44–2.74)	1.00	223	185	0.86
Cough	5	6	0.81 (0.18–3.50)	1.00	7	19	0.11	14	8	2.09 (0.70–6.51)	0.22	118	51	0.79	23	21	1.19 (0.48–2.96)	0.83	167	103	0.54
Skin eruption	3	5	0.57 (0.08–3.18)	0.71	16	20	0.47	6	6	1.00 (0.24–4.10)	1.00	29	31	0.94	10	7	1.55 (0.47–5.34)	0.59	38	56	0.26
Diarrhea	3	0	∞	0.24	3	0	–	6	6	1.00 (0.24–4.10)	1.00	7	12	0.42	9	8	1.16 (0.35–3.86)	1.00	12	15	0.24
Conjunctivits	1	7	0.12 (0.002–1.05)	0.06	2	22	0.40	6	1	6.77 (0.76–318.28)	0.11	19	4	0.80	9	9	1.00 (0.31–3.23)	1.00	18	26	0.18
Vomiting	2	6	0.30 (0.03–1.84)	0.27	2	14	0.25	5	2	2.68 (0.41–29.42)	0.43	15	3	0.37	7	7	1.00 (0.27–3.70)	1.00	18	17	1.00
Anal fissure	1	0	∞	1.00	1	0	–	1	0	∞	1.00	2	0	–	4	1	4.29 (0.40–216.11)	0.36	19	1	0.50
Others	2	3	0.65 (0.05–6.01)	1.00	4	15	0.44	2	3	0.65 (0.05–6.03)	1.00	5	16	0.44	14	16	0.82 (0.31–2.15)	0.82	28	32	0.52

Group A Infants protected from birth onwards

Group B Infants protected from month six onwards

*RR* risk ratio, *CI* confidence interval

^a^Number of infants with at least one episode of respective side effects

^b^Calculated with Fisher’s test

^c^Calculated with Wilcoxon test

### Mothers

The associations between pyrethroid exposure and reported side effects are shown in Table 2. In mothers, headache/dizziness and rhinitis were the most prominent symptoms, followed by skin eruption, pruritus, paresthesia, cough, conjunctivitis and other symptoms; other (rare) symptoms included stomachache, diarrhoea, open wound, ear pain, fatigue, kidney pain and thoracal pain. There were no significant differences in the frequency and duration of these symptoms between the two study groups during the overall follow up period, except that mothers of group A reported significant higher frequency of headache/dizziness compared to group B mothers (p = 0.01).

**Table 2:** Frequency and duration of reported side effects among among mothers of infants of group A (N = 45) and group B (N = 45) of an ITN trial conducted in Burkina Faso

Reported side effects	Week 1							Week 2–4							Week 1–18						
	Frequency^a^		RR (95 % CI)^b^	P-value^b^	Duration of symptom (Days)		P-value^c^	Frequency		RR (95 % CI)	P-value	Duration of symptom (Days)		P-value	Frequency		RR (95 % CI)	P-value	Duration of symptom (Days)		P-value
	A	B			A	B		A	B						A	B			A	B	
Headache/diziness	11	2	6.96 (1.35–67.43)	0.01	27	5	0.55	7	5	1.48 (0.37–6.40)	0.76	44	13	0.22	15	9	2.00 (0.70–5.94)	0.23	79	22	0.43
Rhinitis	5	3	1.75 (0.31–11.94)	0.71	10	14	0.39	5	7	0.67 (0.16–2.74)	0.76	103	143	0.94	12	7	1.97 (0.62–6.61)	0.30	71	49	0.68
Skin Eruption	4	2	2.09 (0.28–24.17)	0.68	19	7	0.51	6	2	3.31 (0.54–34.94)	0.27	43	2	0.11	7	3	2.58 (0.54–16.38)	0.31	77	10	0.15
Pruritus	2	2	1.00 (0.07–14.38)	1.00	7	7	1.00	2	0	∞	0.49	12	0	–	4	2	2.09 (0.28–24.17)	0.68	24	8	0.52
Parestheia	2	2	1.00 (0.07–14.38)	1.00	3	2	1.00	2	0	∞	0.49	12	0	–	2	3	0.65 (0.05–6.01)	1.00	15	3	0.22
Cough	1	0	∞	1.00	1	0	–	5	1	5.50 (0.57–265.92)	0.20	30	5	1.00	6	2	3.31 (0.54–34.94)	0.27	37	6	0.75
Conjunctivits	2	0	∞	0.49	2	0	–	2	1	2.05 (0.10–123.44)	1.00	9	1	0.60	3	3	1.00 (0.13–7.90)	1.00	10	4	0.24
Others	8	5	1.73 (0.45–7.31)	0.55	28	12	0.43	11	9	1.29 (0.43–4.01)	0.80	64	42	0.15	14	11	1.40 (0.50–3.95)	0.64	72	53	0.77

Group A Infants protected from birth onwards

Group B Infants protected from month six onwards

*RR* risk ratio, *CI* confidence interval

^a^Number of mothers with at least one episode of respective side effects

^b^Calculated with Fisher’s tests and RR is based on episode duration

^c^Total number of days (sum) of observed symptom per group

## Discussion

This study reports the findings of an individually randomized trial which specifically investigates the side effects of frequent exposure to low levels pyrethroids (deltamethrin) among ITNs users (both infants and mothers) in a highly endemic malaria area. The results for mothers are different from those for infants. While among the mothers there was a significantly increased risk of the symptom headache/dizziness in those having been exposed to ITNs, no significant differences in the frequency and duration of potential side effects were observed among infants.

Acute toxicity of pyrethroids usually results from occupational use, such as unsafe spraying of the insecticides (e.g. using higher concentration, longer exposure duration, spraying against the wind, or lack of personal protection) [10, 22] or unprotected treatment of large numbers of bed nets with the insecticide [32]. The onset of acute side effects varies from hours to a few days and depends on the way of absorption and dosage [19]. In this study, there were no symptoms attributed to ITN exposure in infants, but headache/dizziness was attributed to ITN exposure in mothers during the first week. This is surprising, as animal experiments found neonatal rats being 4–17 times more vulnerable to the acute toxicity of pyrethroids than adult rats [33, 34].

### Exposure by inhalation

Pyrethroids have a low vapour pressure, therefore, the contribution of inhalation for pyrethroids toxicity was considered to be largely negligible [14, 35]. However, if used in a confined space, concerns about inhalational exposure may still be justified [36]. This concern was not supported by the findings from this study. However, a higher frequency of cough was reported from ITN users in northern Ghana [37].

Rhinitis may also stem from inhalation exposure, affecting the nasal mucosa and causing irritation, and rhinitis was found as a side effect to pyrethroid exposure in an animal model [38]. However, this has not been seen in this study.

### Skin exposure

It has been reported that, compared to adults, infants absorbed relatively higher amounts of deltamethrin through skin contact when sleeping under an ITN [14]. In this study skin eruption occurred for longer periods in children not being exposed to ITNs, also this finding was not significant. It is well known that ITNs provide a physical and a chemical barrier against a variety of household pests, e.g. spiders, fleas, ticks, carpenter ants, bees, cockroaches and bedbugs, which might cause skin infections [39].

It is surprising that such a protective effect of ITNs regarding skin related symptoms was not seen in mothers of this study. In contrast, mothers exposed to ITNs reported (non-significantly) more days of skin symptoms including skin eruption, pruritus and paraesthesia, which supports findings from other research on this topic [20, 40]. These symptoms are usually transient and reversible, and in particular paresthesia is regarded as an acceptable side effect during ITN usage [10].

### Oral exposure

Oral exposure is likely to occur only in young children, not in their mothers. It has been speculated that infants are at risk of absorbing pyrethroids due to their sucking behaviour. In fact, it has been observed in this study that infants frequently take the ITN into their mouth. However, there was no difference between the study groups with regard to diarrhoea and vomiting. Interestingly, in this study, anal fissure was found to be more common in infants exposed to ITNs, also this finding did not reach statistical significance. Anal fissures occur frequently in infants, and constipation is considered the predominant cause [41]. Constipation has also been reported as a potential side-effect related to pesticide contact [42].

### Systemic toxicity

Systemic toxicity may develop after intense dermal exposure, inhalation or ingestion. Symptoms of systemic toxicity include headache/dizziness, convulsion, fatigue and vomiting. These symptoms were partly included into the category “other symptoms” in this study. There were no obvious indications for symptoms of systematic toxicity being associated with ITN exposure in this study. Only headache/dizziness was significantly more frequently reported in mothers exposed to ITNs compared to those not exposed during the first week. This finding is consistent with reports from the literature [43–45].

It appears important to consider a variety of pyrethroid exposure possibilities of mothers. Besides sleeping under an ITN, mothers are also in charge of hanging it up, washing it and re-impregnating it. The bed nets of this trial were regularly re-impregnated (0.4 g deltamethrin, K–O TAB, Aventis) before the beginning of the rainy season and re-impregnation coverage was always over 95 % [46]. This could partly explain the associations between ITN exposure and headache/dizziness in mothers.

This study has strengths and limitations. With regard to its strengths it has to be considered that it is nested into a large randomized controlled trial, making bias less likely. With regard to potential limitations it should be considered that the data on potential side effects are mainly based on self-reports in a rather limited sample size. While significant differences may well have occurred by chance, more rare differences in the occurrence of side effects may have been missed due to lack of power. Moreover it is possible that it may not be culturally acceptable to honestly report adverse effects of a product which has been free of charge which may underestimate potential side effects of the insecticide [37]. Finally, the follow up period was only 18 weeks which makes the detection of long-term effects impossible.

## Conclusions

The study provides further evidence for ITNs being rather safe in children and even in newborns. Given the high impact of ITNs on malaria burden in endemic areas, these findings further support an unrestricted use of ITNs for malaria prevention.
